# Combination of Urine Exosomal mRNAs and lncRNAs as Novel Diagnostic Biomarkers for Bladder Cancer

**DOI:** 10.3389/fonc.2021.667212

**Published:** 2021-04-27

**Authors:** Haiming Huang, Jialin Du, Bo Jin, Lu Pang, Nan Duan, Chenwei Huang, Jiayin Hou, Wei Yu, Han Hao, Haixia Li

**Affiliations:** ^1^ Department of Clinical Laboratory, Peking University First Hospital, Beijing, China; ^2^ Department of Urology, Peking University First Hospital and Institute of Urology, Beijing, China

**Keywords:** urine exosomes, mRNA, lncRNA, non-invasive biomarkers, diagnosis, bladder cancer

## Abstract

**Background:**

The recent discovery of miRNAs and lncRNAs in urine exosomes has emerged as promising diagnostic biomarkers for bladder cancer (BCa). However, mRNAs as the direct products of transcription has not been well evaluated in exosomes as biomarkers for BCa diagnosis. The purpose of this study was to identify tumor progression-related mRNAs and lncRNAs in urine exosomes that could be used for detection of BCa.

**Methods:**

RNA-sequencing was performed to identify tumor progression-related biomarkers in three matched superficial tumor and deep infiltrating tumor regions of muscle-invasive bladder cancer (MIBC) specimens, differently expressed mRNAs and lncRNAs were validated in TCGA dataset (n = 391) in the discovery stage. Then candidate RNAs were chosen for evaluation in urine exosomes of a training cohort (10 BCa and 10 healthy controls) and a validation cohort (80 BCa and 80 healthy controls) using RT-qPCR. The diagnostic potential of the candidates were evaluated by receiver operating characteristic (ROC) curves.

**Results:**

RNA sequencing revealed 8 mRNAs and 32 lncRNAs that were significantly upregulated in deep infiltrating tumor region. After validation in TCGA database, 10 markedly dysregulated RNAs were selected for further investigation in urine exosomes, of which five (mRNAs: KLHDC7B, CASP14, and PRSS1; lncRNAs: MIR205HG and GAS5) were verified to be significantly dysregulated. The combination of the five RNAs had the highest AUC to disguising the BCa (0.924, 95% CI, 0.875–0.974) or early stage BCa patients (0.910, 95% CI, 0.850 to 0.971) from HCs. The expression levels of these five RNAs were correlated with tumor stage, grade, and hematuria degrees.

**Conclusions:**

These findings highlight the potential of urine exosomal mRNAs and lncRNAs profiling in the early diagnosis and provide new insights into the molecular mechanisms involved in BCa.

## Introduction

Bladder cancer (BCa) is the tenth most common cancer type worldwide (1). The incidence of BCa is approximately four times higher in men than in women, and BCa mostly affects older people (2). At diagnosis, approximately 75% of newly diagnosed patients of BCa are non–muscle invasive bladder cancer (NMIBC), which is associated with a 5-yr survival of 90% (3). Nevertheless, 15% of patients will progress to muscle-invasive bladder cancer (MIBC) within 5 years, which is characterized by a high progression and metastasis rate. And the 5-yr survival rate for patients with metastasis MIBC is only 5% (4). Therefore, effective methods for early diagnosis are imperative to reduce death. A combination of cytology and cystoscopy remains the gold standard to diagnosis of BCa, whereas cystoscopy is invasive and urine cytology is limited by its low sensitivity (20–53%), especially in low-grade tumor (5). Moreover, BCa as a molecularly heterogeneous disease that is characterized by genomic instability and a high somatic mutation rate (6), single tumor biopsy may vastly underestimate the heterogeneity of the whole tumor (7). Some urine tumor markers, such as the nuclear matrix protein 22 (NMP22) (8) and bladder tumor antigen (BTA) (9) have been approved by The Food and Drug Administration (FDA) for BCa diagnosis. However, due to their moderate assay performance, high cost and may be falsely elevated in benign conditions (10), widespread adoption of such assays has not been occurred. Therefore, it is urgently necessary to develop innovative and noninvasive diagnostic biomarkers that have high sensitivity and specificity.

Exosomes are extracellular vehicles (EVs) with a size range of 40–150 nm in diameter with an endosomal origin; small EVs are actively secreted by cells into biofluids, including blood and urine (11–13). Exosomes can contain many constituents of a cell, including nucleic acids and proteins (11). RNA with exosomes are promising for cancer detection because they are highly representative of their cell of origin (13), and provide protection for RNA during sample processing (14). The recent discovery of miRNAs and lncRNAs in urine exosomes have emerged as promising diagnostic biomarkers for BCa. For example, the level of urine exosomal miR-21-5p was overexpressed in urine exosomes from urothelial carcinoma patients with negative urine cytology and could be used as a novel biomarker for urothelial carcinoma (AUC = 0.900; sensitivity = 75.0%; specificity = 95.8%) (15). Moreover, most recent studies have used a miRNAs or lncRNAs panel to improve the accuracy of BCa diagnosis. A quantitative reverse transcription PCR analysis revealed that the levels of three exosomal lncRNAs (MALAT1, PCAT-1, and SPRY4-IT1) were higher in BCa urine samples than in healthy control samples, with a sensitivity and specificity of 70.2 and 85.6% (AUC = 0.854) (16). Similarly, analyses of a panel of seven miRNAs in urine samples displayed an AUC value of 0.923, the corresponding sensitivities of this panel for the detection of BCa stages Ta, T1, and T2-T4 were 86.4%, 93.0%, and 97.8%, respectively (17). However, exosomal research in cancer, especially BCa, is still in the early stages. mRNA as the direct products of transcription has not been well evaluated in exosomes as biomarkers for BCa diagnosis.

In this study, we firstly performed an RNA sequencing (RNA-seq) to identify the tumor progression related mRNAs and lncRNAs between three matched superficial and deep infiltrating tumor regions of MIBC. Then we analyzed the expressions profiles of dysregulate RNAs in urinary exosomes in two independent cohorts of BCa patients. For the first time, a panel of combined mRNA and lncRNA was identified as an effective diagnostic tool for BCa. Finally, we explored the progression features of the RNAs in our panel and hypothesized that these mRNAs and lncRNAs may play important biological roles in the initiation of muscle invasion.

## Materials and Methods

### Study Design and Subjects

**Figure 1:**
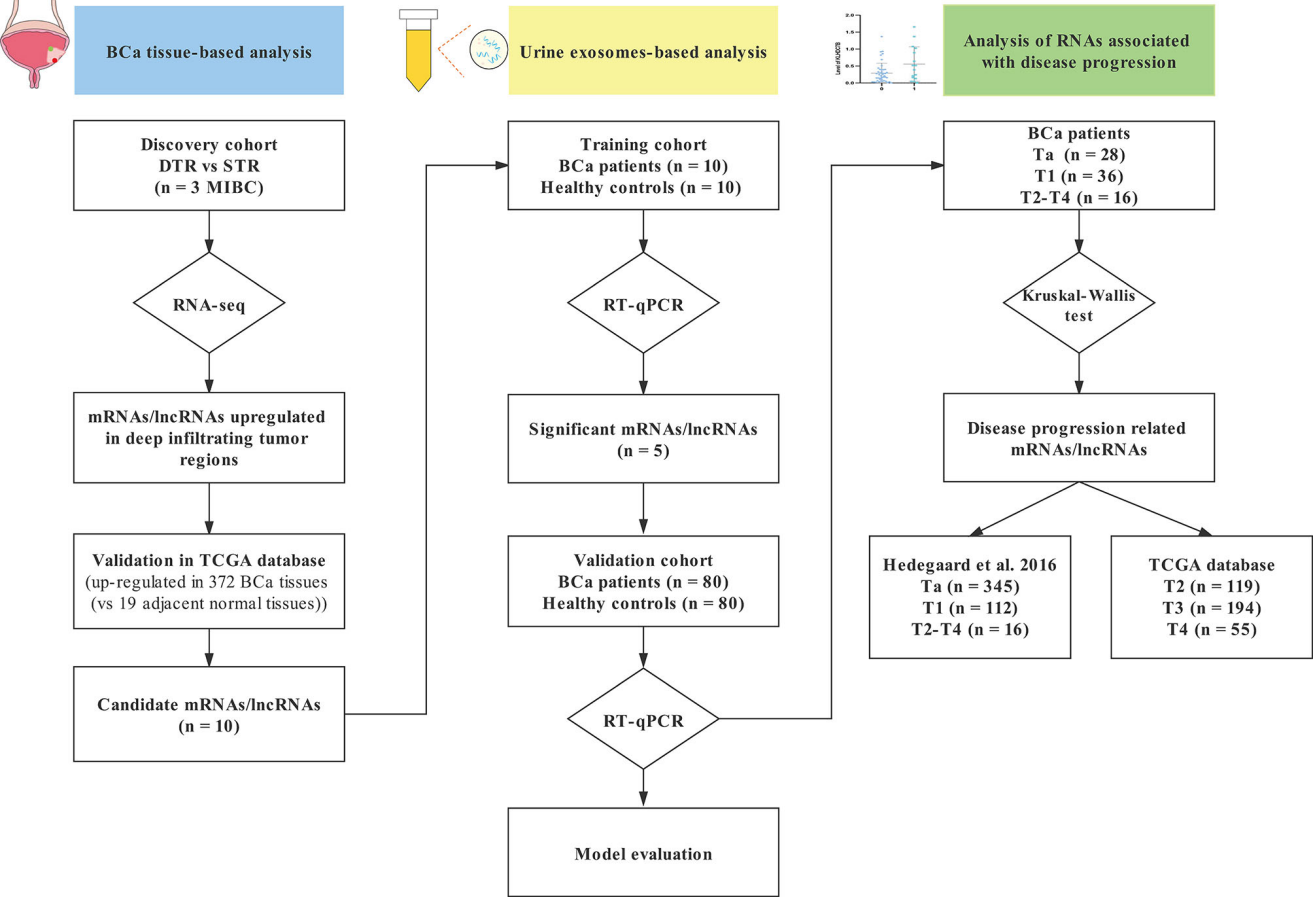
A flowchart of research design. *STR*, superficial tumor regions; *DTR*, deep infiltrating tumor regions; *TCGA*, The Cancer Genome Atlas database; *BCa*, bladder cancer; *RT-qPCR*, real-time quantitative PCR.

The study included three progressive stages: the discovery stage, the training stage, and the validation stage, and the flowchart was shown in **Figure 1**.

In the discovery stage, we performed RNA-seq on superficial tumor and deep infiltrating tumor regions of three matched MIBC specimens obtained from radical cystectomy at Department of Urology in Peking University First Hospital between October 2019 and February 2020 to initially identify dysregulated mRNAs and lncRNAs that may be involved in tumor progression. To verify whether these molecules are tumor-related, we used The Cancer Genome Atlas (TCGA) database as an independent validation group to compare the expression of these molecules in BCa tissues and adjacent normal tissues. The biomarkers collected complied with the criteria as follows: (1) to ensure that candidate RNAs are tumor-related and can be detected in urine exosomes, especially in patients with early stage BCa, dysregulated RNAs obtained by RNA-seq were upregulated in deep infiltrating tumor regions as previously described (18); (2) the average expression of candidate RNAs were between 3 and 50 to avoid biomarkers concentration that were too low to be detected in urine exosomes or housekeeper genes rather than tumor-related genes; (3) candidate biomarkers were upregulated in BCa tissues compared to adjacent normal tissues in TCGA cohort, P < 0.05.

Then, candidate RNAs were further measured by real-time quantitative PCR (RT-qPCR) in exosomes derived from 20 paired [10 BCa, 10 healthy controls (HCs)] urine samples during the training stage. We considered an RNA to be significantly changed between the two groups if it met the following criteria: threshold cycle (Ct) value <35, and detection rate >75%, as previously described (19). Furthermore, we examined the significantly changed urine RNAs in a validation cohort with 80 BCa patients and 80 HCs.

The BCa patients were enrolled from the Department of Urology in Peking University First Hospital between October 2019 and December 2020. All patients were confirmed through histological examination of biopsy samples, and no neoadjuvant treatment or radiotherapy was received prior to tissue/urine collection. The tumors were staged according to the 8th Union of International Control of Cancer (UICC) classification (20). **Table 1** summarizes the clinicopathological characteristics of the BCa patients enrolled in the training and validation stages, including sex, age, hematuria degrees, tumor stage, and tumor grade. The HCs were randomly selected from participants enrolled in healthy check-up programs that were conducted in the Department of Clinical Laboratory in Peking University First Hospital and showed no disease. The demographic information of the HCs was also available in **Table 1**. The study was approved by Peking University First Hospital Ethics Committee. Informed consent was obtained by all the participated patients.

**Table 1:** Demographic and clinical characteristics in training and validation cohorts.

Variable	Training cohort			Validation cohort		
	HCs (n = 10)	BCa (n = 10)	P value	HCs (n = 80)	BCa (n = 80)	P value
**Sex**			0.845			0.063
Male	8 (80%)	9 (90%)		56 (70%)	66 (82.5%)	
Female	2 (20%)	1 (10%)		24 (30%)	14 (17.5%)	
**Age ± SD (years)**	45.7 ± 14.0	61.4 ± 10.5	<0.001	47.4 ± 11.3	64.8 ± 12.5	<0.001
**Hematuria degrees**						
Negative/trace		4 (40%)			31 (38.75%)	
+/++		4 (40%)			26 (32.5%)	
+++		2 (20%)			23 (28.75%)	
**Tumor Stage**						
pTa		5 (50%)			28 (35.0%)	
pT1		4 (40%)			36 (45.0%)	
pT2–pT4		1 (10%)			16 (20.0%)	
**Tumor Grade**						
Low		4 (40%)			35 (43.75%)	
High		6 (60%)			45 (56.25%)	

BCa, bladder cancer; HCs, healthy controls.

Data are given as n (%) unless otherwise noted.

The cohorts of Hedegaard et al. (n = 473) and TCGA database (n = 391) were used as our study validation cohort regarding NMIBC and MIBC, respectively (21). Hedegaard et al. performed paired-end whole transcriptome, strand-specific RNA-seq (Illumina HiSeq platform) of a cohort consisted of 457 NMIBC (Ta: 345, T1: 112) and 16 MIBC samples. TCGA cohort consisted of 4 NMIBC, 368 MIBC (T2: 119, T3: 194, T4: 55) and 19 adjacent normal samples, and transcriptome expression profiles generated by paired-end whole transcriptome RNA-seq (Illumina HiSeq platform). Clinical and normalized expression data were downloaded for Hedegaard et al. cohort by EMBL-EBI ArrayExpress (accession number ArrayExpress: E-MTAB-4321; https://www.ebi.ac.uk/arrayexpress/experiments/E-MTAB-4321/) and for TCGA cohort by UCSC Xena (https://xenabrowser.net/datapages/).

### High-Throughput RNA-Seq Analysis

High-throughput RNA-seq analysis was conducted by OE Biotechnology Co., Ltd (Shanghai, China). In brief, total RNAs were isolated separately from three matched MIBC tissues with superficial regions and deep infiltrating tumor regions by using the TRIzol reagent (Invitrogen, Carlsbad, CA, USA). Then, the RNA quality and concentration were evaluated using an Agilent 2100 Bioanalyzer (Agilent Technologies, Santa Clara, CA, USA) and a ND1000 spectrophotometer (NanoDrop, Wilmington, DE, USA), respectively. A total of 5 μg RNA per sample was used for the preparations. After ribosomal RNA (rRNA) was digested by TruSeq Stranded Total RNA with Ribo-Zero Gold Kit (Illumina, San Diego, CA, USA), the cDNA library was constructed using the NEBNext Ultra RNA Library Prep Kit for Illumina (NEB, Ipswich, MA, USA). The libraries were sequenced on an Illumina HiSeq X Ten platform (Illumina, San Diego, CA, USA) in accordance with the manufacturer’s instructions. The fragments per kilobase of model per million base pairs sequenced (FPKM) was used to calculate the expression levels of mRNA or lncRNA. Using the estimateSizeFactors function of the DESeq R package (version 1.8.3) to normalize the counts, and using nbinomTest function to calculate P value and foldchange (FC) values for the difference comparison. Finally, the differential expression (DE) RNAs with P < 0.05 and FC > 2 were identified. The function and biological pathways of DE RNAs were analyzed with Gene Ontology (GO) (http://www.geneontology.org) and Kyoto Encyclopedia of Genes and Genomes (KEGG) (http://www.genome.ad.jp/kegg/) databases by using Hypergeometric Distribution Test. P value <0.05 were considered to be significantly enriched. More details are provided in the **Supplementary Methods**.

### Urine Collection and Preparation

Ten ml of urine was initially centrifuged at 2,000 g for 10 min to remove particles and debris, and then the supernatant was further centrifuged at 10,000 rpm for 15 min to completely remove cell debris, and the supernatant fluids were then collected and stored at −80°C until exosome extraction. All the experiments were finished within 2 h.

### Exosome Characterization

Exosomes were isolated from urine samples using a commercial kit (Norgen Biotek Corp, Product No. 47200, Canada) for Nanoparticle Tracking Analysis, Transmission Electron Microscopy and Western blot analysis.

### Nanoparticle Tracking Analysis (NTA)

The size distribution and concentration of exosomes were determined using NTA. Briefly, exosomes extracted from urine samples were first diluted in 1 ml phosphate buffered saline (PBS) and mixed well, and then the diluted exosomes were injected into the ZetaView particle tracker (ZetaVIEW S/N 17-310, Particle Metrix, Germany). The ZetaView software (version 8.04.02) was used to analyze the data. Filtered PBS was used as a control.

### Transmission Electron Microscopy (TEM)

Exosomes extracted from urine samples were first resuspended in 100 ul PBS, and then a 20 µl of exosomes was applied to a glow discharged 200-mesh Cu grid coated with carbon-Formvar film (ProSciTech, Kirwan, QLD, Australia) and allowed to absorb for 1 min. Finally, exosomes were stained with 20 µl 2% uranyl acetate at room temperature for 1 min and then dried using an infrared lamp for 10 min. Samples were imaged using a JEM-1400 transmission electron microscope (JEOL Inc., Peabody, MA, USA) to observe the morphologies and sizes of the exosomes.

### Western Blot

The 4x NuPAGE LDS Sample Buffer (Thermo Fisher Scientific, MA, USA) was diluted 1:3 with RIPA lysis buffer (Solarbio, China) to 1x NuPAGE LDS Sample Buffer. Then, exosomes extracted from urine samples were first resuspended in 100 µl 1x NuPAGE LDS Sample Buffer and incubated for 10 min at 37°C, 10 min at 60°C, and 5 min at 95°C. An aliquot of 20 µl of each protein preparation described above was loaded and separated under non-reducing conditions onto sodium dodecyl sulfate–polyacrylamide gel electrophoresis (10%, SDS-PAGE), followed by transferring to the polyvinylidene fluoride (PVDF, Millipore, USA) membranes. After being blocked with tris buffered saline tween (TBST) buffer containing 5% non-fat milk for 1 h at room temperature, the PVDF membranes were incubated with primary antibodies anti-CD9 antibody (rabbit IgG) (Ab92726, 1:1,000, Abcam, UK) and anti-TSG101 (mouse IgG) (Ab83, 1:1,000, Abcam, UK) at 4°C overnight. Next, the PVDF membranes were incubated with goat anti-rabbit IgG HRP-linked secondary Antibody (7074P2, 1:3,000, Cell Signaling Technology, China) and goat anti-mouse HRP secondary antibody (ZB-2305, 1:5,000, ZSGB-BIO, China). Finally, chemiluminescent signals were detected using Immobilon Western Chemiluminescent HRP Substrate (WBKLS0100, Millipore, USA), and imaging was performed on the G:BOX Chemi XT4 (Syngene, UK).

### Isolation of Total RNA From Exosomes in Urine

Total exosome RNA was extracted from the urine samples using the Urine Exosome RNA Isolation Kit (Norgen Biotek Corp, Product No. 47200, Canada) according to the manufacturer’s instructions and evaluated by a NanoDrop spectrophotometer (Thermo Fisher Scientific, USA). Briefly, Frozen urine samples were thawed on ice until samples were completely liquid, then centrifuged at 2,500 rpm for 10 min to remove any residual cellular debris. Next, 300 μl of Slurry B1 was added to 5 ml of urine sample, incubated for 15 min at room temperature, and mixed well by vortexing and centrifuged the sample at 2,500 rpm for 15 min to pellet the resin and decant the supernatant. The pellet was resuspended in 300 μl of lysis buffer A and incubated for 15 min at room temperature. Next, 300 µl 67% isopropanol was added to the mixture and vortexed for 1 min. The lysate was then transferred to a mini filter spin column assembled with a collection tube and centrifuged at 14,000 rpm for 2 min and decant flowthrough. Next, 400 µl of wash solution A was applied to the column and again centrifuged at 14,000 rpm for 1 min. The wash step was done three times, and 80 µl of elution solution A was applied to the column and centrifuge for 3 min at 2,000 rpm, followed by 2 min at 14,000 rpm.

### Real-Time Quantitative PCR (RT-qPCR)

To validate mRNAs and lncRNAs identified by RNA sequencing, 320 ng total RNA was added to a final volume of 40 μl mixed reagent for reverse transcription. cDNA was synthesized using PrimeScript reverse transcriptase Master Mix kit (RR036A, TaKaRa, Dalian, China). The reaction mixture was incubated at 37°C for 30 min, followed by 85°C for 5 s and 4°C for 60 min. Real-time PCR was performed using a SYBR^®^ Premix Ex Taq™ II (RR820A, Takara, Dalian, China) on an ABI 7500 real-time PCR system (Applied Biosystems, CA, USA). The PCR cycles were set at 95°C for 30 s, followed by 40 cycles at 95°C for 5 s, annealing/extension at 60°C for 34 s. The primers were designed by Shanghai Sangon Company (Shanghai, China). Glyceraldehyde-3-phosphate dehydrogenase (GAPDH) was used as the endogenous control for mRNA or lncRNA, respectively. The comparative cycle threshold (2^−ΔCt)^ method was used to analyze relative expression levels. The primers were listed in **Supplementary Table S1**.

### Statistical Analysis

The differences in the expressions of selected mRNAs and lncRNAs between BCa tumor tissues (n = 372) and adjacent normal tissues (n = 19) in TCGA database were assessed by non-parametric Mann-Whitney U test. The false discovery rate (FDR) control method (22) was used in multiple hypothesis testing to correct for P values, the adjusted P-value <0.05 was considered statistically significant. The differences in the expressions of urine exosomes derived RNAs between BCa patients and HCs were assessed by non-parametric. Mann-Whitney U test. The chi-square test and student’s t test were used to determine differences in demographics between the two groups. Multiple comparisons were performed using Kruskal-Wallis test.

To validate dysregulate candidate urine exosomal RNAs as independent biomarkers for BCa, multiple regression analyses were performed to adjust for the influence of confounding factors on the incidence of BCa. Receiver operating characteristic (ROC) curves were used to evaluate the diagnostic power of the candidate exosomal RNAs for BCa. Each candidate exosomal RNAs accuracy for BCa was assessed by the area under the curve (AUC), sensitivity, and specificity based on ROC curve analyses. The Youden index was used to determine optimal cut-off value. The logistic regression model was used to generate a predictive value by combing the exosomal RNAs. Survival curves were plotted using Kaplan-Meier method, and significant differences between the curves were determined using log-rank test. Optimal cut-off values of the candidate RNAs expression levels in Hedegaard et al. and TCGA cohorts were determined using the ROC curves. Cox regression analyses were used to identify independent prognostic factors for progression prediction. Spearman’s rank correlation tests were carried out to analyze the relationships between the candidate RNAs and clinical parameters. All of these analyses were performed on SPSS 24.0 software (IBM Corp., Armonk, NY, USA). A P-value <0.05 was considered statistically significant. Scatter diagrams and Kaplan-Meier curves were made with GraphPad Prism 5 (San Diego, CA, USA). Data were shown as mean ± SD. ROC curves were constructed using the DeLong model (MedCalc Software bvba 18.9, Ostend, Belgium).

## Results

### Identification of mRNA and lncRNA Profiles *via* RNA-Seq in Different Regions of MIBC

**Figure 2:**
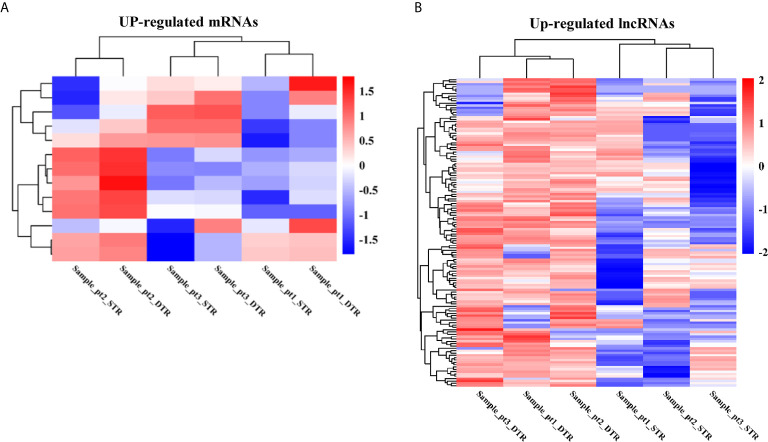
The heatmap of significantly upregulated mRNAs **(A)** and lncRNAs **(B)** in deep infiltrating tumor regions (DTR) compared with the superficial tumor regions (STR) of MIBC.

To assess dysregulated mRNAs and lncRNAs in multiple regions of MIBC, we conducted RNA sequencing between three matched superficial tumor regions and deep infiltrating tumor regions of MIBC. A total of 13 mRNAs and 132 lncRNAs were upregulated in deep infiltrating tumor regions compared with superficial tumor regions (FC > 2 and P < 0.05) (**Figures 2A, B**).

**Figure 3:**
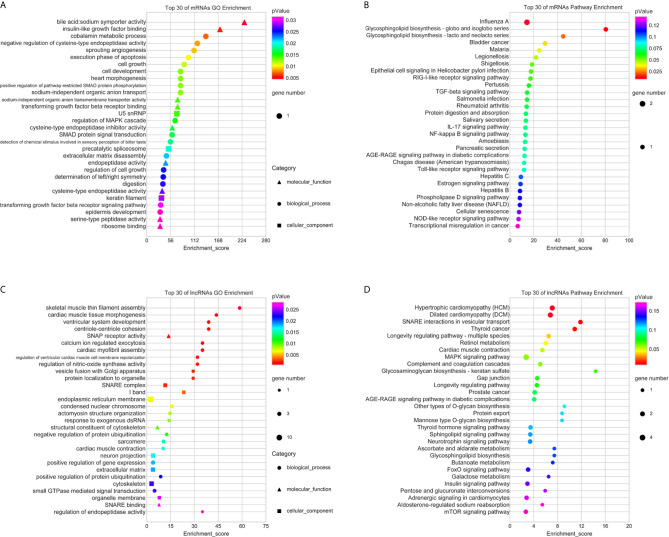
GO and KEGG pathway analyses of upregulated mRNAs and lncRNAs. The top 30 of GO terms enriched in upregulated mRNAs **(A)** and lncRNAs **(C)**; the top 30 of KEGG pathways enriched in upregulated mRNAs **(B)** and lncRNAs **(D)**.

Then, the GO analyses and KEGG analyses were used to explore the potential biological roles of the differentially expressed RNAs. According to GO analyses, up-regulated mRNAs were enriched in anion transport, cell growth, and regulation of MAPK cascade (**Figure 3A**); upregulated lncRNAs were mainly associated with soluble NSF attachment protein receptor (SNARE), such as SNAP receptor activity, SNARE complex, and SNARE binding; moreover, these lncRNAs were also distribute in endoplasmic reticulum membrane, extracellular matrix, and organelle membrane, which are related to intercellular vesicle transport and signal transduction (**Figure 3C**). Furthermore, the top 30 pathways identified by KEGG pathway analyses were shown in **Figures 3B, D**. Upregulated RNAs were mainly enriched in pathways in cancer, such as bladder cancer, RIG-I-like receptor signaling pathway, TGF-beta signaling pathway, MAPK signaling pathway, FoxO signaling pathway, and mTOR signaling pathway.

Eight mRNAs and 32 lncRNAs displayed average expression were between 3 and 50 in at least one tumor region (**Supplementary Tables S2, S3**). Then, we selected the above mRNAs and lncRNAs for further analysis in TCGA cohort, 12 RNAs (5 mRNAs: KLHDC7B, CASP14, ESM1, PRSS1, and CST1; 7 lncRNAs: GAS5, SNHG12, MIR205HG, PVT1, LINC02474, UCA1, and TUG1) were upregulated in BCa tumor tissues (n = 372) compared to adjacent normal tissues (n = 19) (P < 0.05) were selected as candidate molecules (**Supplementary Tables S2, S3**). In addition, lncRNAs such as UCA1 and TUG1 have been previously reported by other studies were excluded from this study, and the expressions of these two lncRNAs in urine exosomes showed no difference between BCa patients and HCs (16, 23).

### Characterization of Urine Exosomes

**Figure 4:**
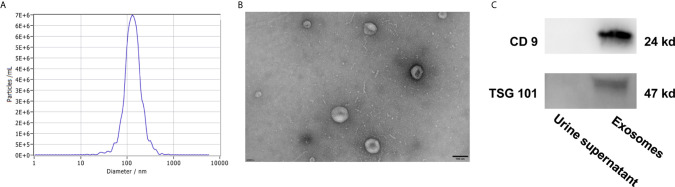
Characterization of urine exosomes by Nanoparticle Tracking Analysis **(A)**, Transmission Electron Microscopy **(B)**, and western blot **(C)**.

To identify the quality and purity of the exosomes we extracted from the urine, NTA analysis were performed to assess the size distribution of the exosomes. As shown in **Figure 4A**, most of the particles we extracted were ∼128.4 nm in diameter, 80% of particles are ranging from 78.1 nm to 212.6 nm in diameter. TEM revealed that they were spherical particles with a cup-shaped morphology around 100 nm (**Figure 4B**), which supported the NTA results. In addition, western blot analysis of protein extracts from these small vesicles, exosomal markers CD9 and TSG101 were positive in the exosomes samples but not in the urine supernatant (**Figure 4C**). Taken together, these results suggested that exosomes were successfully prepared and characterized for downstream applications.

### Evaluation of Candidate mRNAs and lncRNAs in Urine Exosomes

In the training stage, the candidate mRNAs/lncRNAs revealed in discovery cohort were assessed by RT-qPCR in urine exosomes in an independent cohort consisting of 10 BCa patients and 10 HCs. Differentially expressed urine exosomal mRNA KLHDC7B, CASP14, and PRSS1 and lncRNA MIR205HG and GAS5 were selected for further validation (**Supplementary Tables S4, S5**).

**Figure 5:**
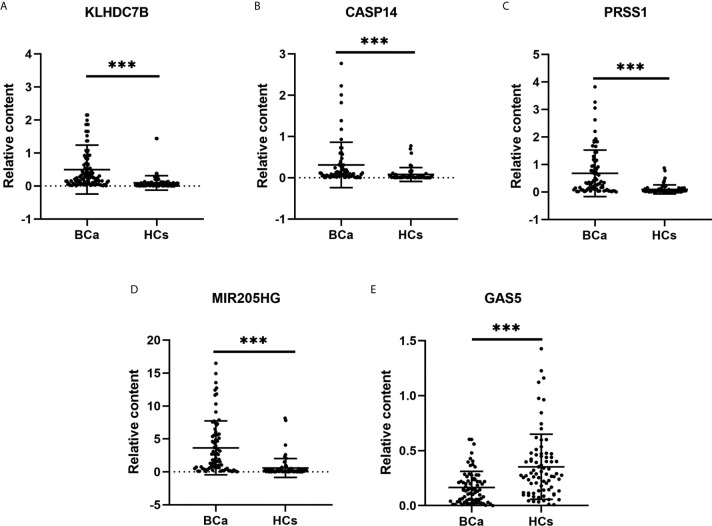
Expression levels of urine exosomal mRNAs and lncRNAs in the validation cohort. **(A–C)**: Expression levels of mRNAs KLHDC7B **(A)**, CASP14 **(B)**, and PRSS1 **(C)** in BCa patients (n = 80) and HCs (n = 80) using RT-qPCR; **(D, E)**: Expression levels of lncRNAs MIR205HG **(D)** and GAS5 **(E)** using RT-qPCR. ***P < 0.001.

To confirm the diagnostic value of our selected RNAs in urine exosomes of BCa patients, we validated our results in a larger population (80 BCa patents and 80 HCs). Significantly higher expression of exosomal KLHDC7B, CASP14, and PRSS1 and MIR205HG were observed in BCa patients compared with the HCs (all P < 0.001) (**Figures 5A–D**). Meanwhile, lower expression of exosomal GAS5 was detected in the BCa patients (P < 0.001) (**Figure 5E**).

### Diagnostic Performance of Five Exosomal RNAs to Detect BCa

To validate dysregulate candidate exosomal RNAs as independent biomarkers for BCa, we performed multiple regression analyses to adjust for the influence of confounding factors on the incidence of BCa, such as age, which was significantly different between BCa patients and HCs (P < 0.001) (**Table 1**). The result showed that a higher level of exosomal KLHDC7B (odds ratios, OR = 1.004, 95% CI: 1.001–1.007, P = 0.006), CASP14 (OR = 1.002, 95% CI: 1.000–1.005, P = 0.046), PRSS1 (OR = 1.003, 95% CI: 1.001–1.005, P = 0.003), and MIR205HG (OR = 1.554, 95% CI: 1.221–1.976, P < 0.001) were associated with increased BCa risk (**Supplementary Tables S6**). However, lower level of exosomal GAS5 (OR = 0.036, 95% CI: 0.003–0.387, P = 0.006) was associated with increased BCa risk (**Supplementary Tables S6**). Hence, exosomal KLHDC7B, CASP14, PRSS1, and MIR205HG were independent risk factors in BCa patients. Whereas, exosomal GAS5 may be regarded as an independent protective factor in patients with BCa.

**Figure 6:**
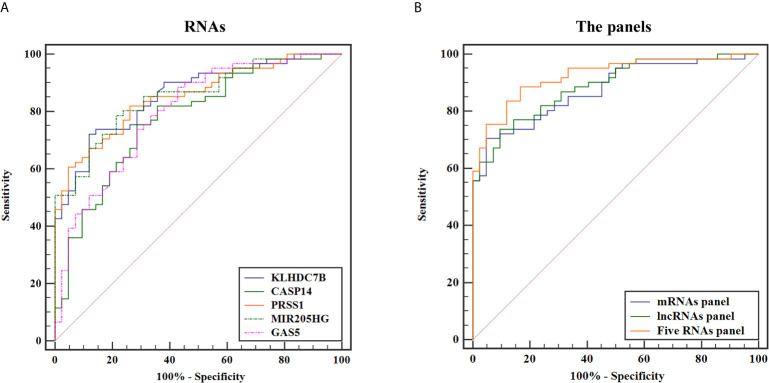
ROC curves for discriminating BCa patients from HCs based on urine exosomal KLHDC7B, CASP14, PRSS1, MIR205HG, and GAS5 **(A)** alone and in combination **(B)**.

To evaluate diagnostic performance of the identified mRNAs and lncRNAs for BCa detection, ROC curve analyses were carried out using the data generated in the validation cohort. As shown in **Figure 6A**, the diagnostic accuracy of exosomal KLHDC7B, CASP14, PRSS1, MIR205HG, and GAS5 measured by AUC were 0.842 (95% CI = 0.775 to 0.908), 0.765 (95% CI = 0.678 to 0.852), 0.823 (95% CI = 0.754 to 0.891), 0.843 (95% CI = 0.779 to 0.906), and 0.729 (95% CI = 0.649 to 0.808) compared to HCs, respectively (**Table 2**). Meanwhile, on combining three exosomal mRNAs (KLHDC7B, CASP14, and PRSS1) by logistic regression model, the mRNAs panel exhibited an AUC of 0.880 (95% CI = 0.818 to 0.943) (**Figure 6B**) (**Table 2**). Similarly, the combined panel of two exosomal lncRNAs (MIR205HG and GAS5) displayed an AUC of 0.842 (95% CI = 0.777 to 0.906) (**Figure 6B**) (**Table 2**). However, using DeLong test, we found that the diagnostic efficiency of the exosomal mRNAs/lncRNAs combination panel was not significantly improved compared with the diagnostic efficiency of single molecules such as KLHDC7B, PRSS1, or MIR205HG (P > 0.05) (**Supplementary Table S7**).

**Table 2:** The performance of ROC curve analysis, PPV, NPV, and accuracy for each RNAs and the combined RNAs panel in validation cohort.

RNAs	AUC	95% CI	P value	Sensitivity%	Specificity%	PPV%	NPV%	Accuracy%
**KLHDC7B**	0.842	0.775–0.908	<0.001	68.5	88.3	68.5	86.7	76.7
**CASP14**	0.765	0.678–0.852	<0.001	77.5	70.6	80.3	64.7	73.8
**PRSS1**	0.823	0.754–0.891	<0.001	78.1	75.0	60.3	87.5	73
**MIR205HG**	0.843	0.779–0.906	<0.001	77.3	83.1	56	88.7	71.9
**GAS5**	0.729	0.649–0.808	<0.001	78.7	60.3	74.7	64.1	69.3
mRNAs panel^a^	0.88	0.818–0.943	<0.001	71.9	95.2	75	83.3	78.3
lncRNAs panel^b^	0.842	0.777–0.906	<0.001	67.1	87.1	70	80	75
Five RNAs panel^c^	0.924	0.875–0.974	<0.001	88.5	83.3	83.6	88.1	85.4

ROC, receiver operating characteristic; PPV, positive predictive value; NPV, negative predictive value; AUC, area under the receiver operating characteristic curve; CI, confidence interval.

a mRNAs panel: KLHDC7B, CASP14, PRSS1.

b lncRNAs panel: MIR205HG, GAS5.

c Five RNAs panel: KLHDC7B, CASP14, PRSS1, MIR205HG, GAS5.

Furthermore, the diagnostic performance of the combination of exosomal mRNAs and lncRNAs was calculated, the predictive probability of being diagnosed with BCa was calculated using equation as follows: Logit(*P*) = 4.003 × KLHDC7B-3.717 × CASP14 + 7.788 × PRSS1 + 0.199 × MIR205HG − 6.927 × GAS5 + 0.330. The highest AUC of 0.924 (95% CI, 0.875 to 0.974) was obtained and the sensitivity and specificity were correspondingly increased to 88.5 and 83.3% respectively (**Figure 6B**) (**Table 2**). The diagnostic efficiency of five exosomal RNAs panel was not only significantly higher than that of any single molecule, but also higher than that of the combination of exosomal mRNAs/lncRNAs panel (all P < 0.05) (**Supplementary Tables S7**). Therefore, the combination of exosomal mRNAs and lncRNAs could serve as a potential non-invasive biomarker for the diagnosis of BCa.

### Diagnostic Accuracy of Five Exosomal RNAs Panel in Distinguishing Early-Stage BCa Cases and HCs

**Figure 7:**
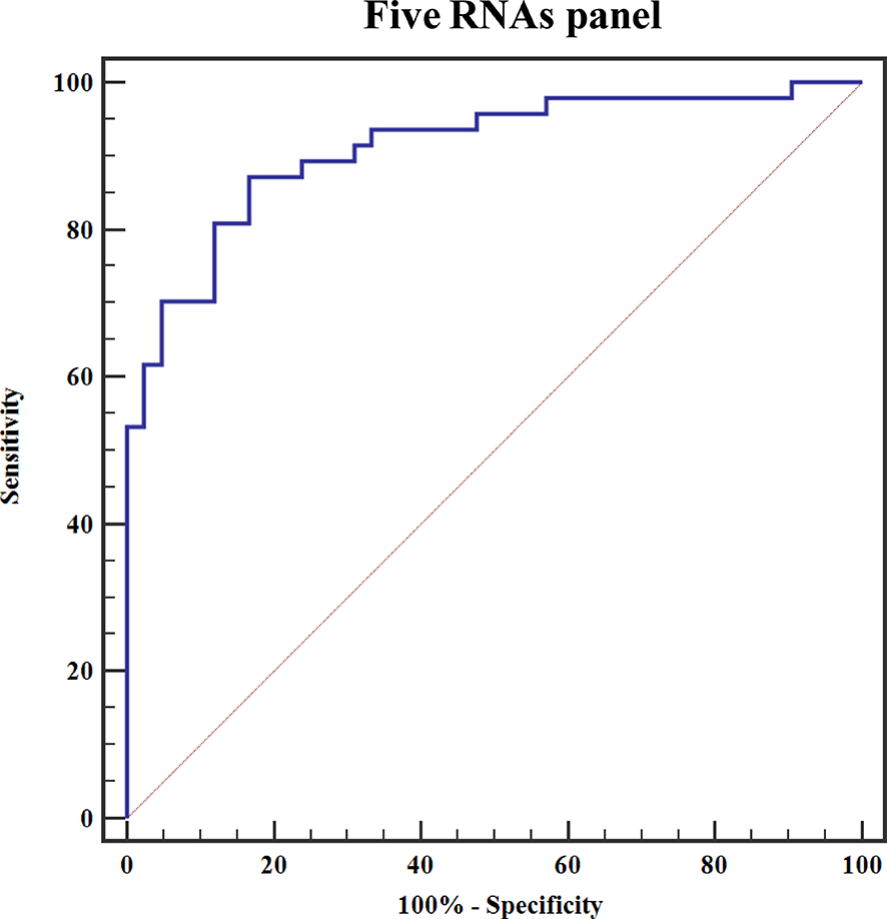
ROC curve for discriminating early-stage BCa patients from HCs based on the constructed five exosomal RNAs panel.

To elucidate the early-stage BCa diagnosis efficacy of five exosomal RNAs, we analyzed the expression of these five RNAs in the urine exosomes of patients with NMIBC [n = 64 (Ta = 28, T1 = 37)] in the validation cohort. Expression levels of exosomal KLHDC7B, CASP14, PRSS1, and MIR205HG were significantly increased in early BCa patients as compared to the HCs (all P < 0.001) (**Supplementary Figures S1A–D**), and the expression of exosomal GAS5 was significantly decreased in patients with early BCa compared to that in HCs (P < 0.001) (**Supplementary Figure S1E**). Next, ROC curve was employed to evaluate the performance of the constructed five exosomal RNAs panel. The combination panel displayed a strong diagnostic accuracy for early-stage BCa, with an AUC of 0.910 (95% CI, 0.850 to 0.971, sensitivity = 87.2% and specificity = 83.3%; PPV = 80.9%, NPV = 88.1%) (**Figure 7**).

### The Levels of Five Exosomal RNAs in Different Tumor Stages

**Figure 8:**
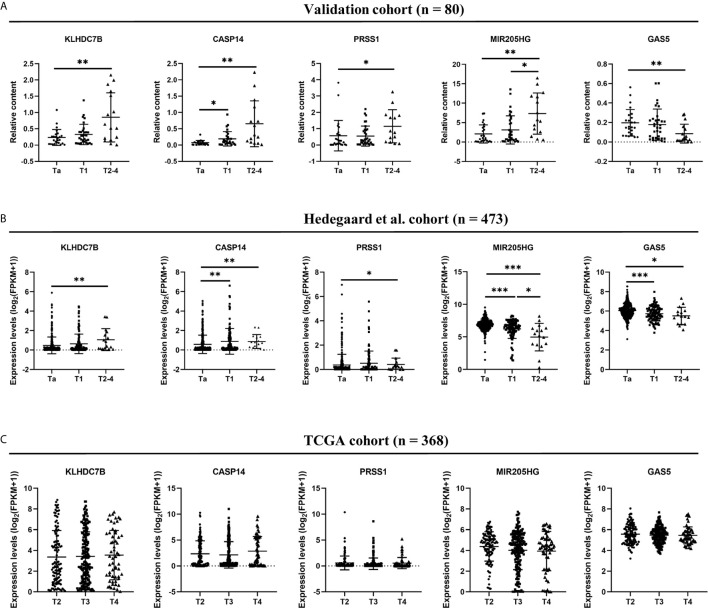
Comparison of five RNAs expression levels among different BCa stage in urine exosomes **(A)**, Hedegaard et al. (24) validation cohort **(B)**, and TCGA validation cohort **(C)**. *P < 0.05, **P < 0.01, ***P < 0.001.

To analyze whether these five RNAs were associated with tumor progression, we analyzed the expression levels of five exosomal RNAs between BCa patients with different tumor stages in the validation cohort. As shown in **Figure 8A**, significantly higher exosomal KLHDC7B, CASP14, PRSS1, and MIR205HG levels (all P < 0.05) were expressed in muscle-invasive (T2–T4) compared to superficial Ta tumors. Meanwhile, significantly lower levels of exosomal GAS5 was observed in the advanced BCa (T2–4) (P < 0.05).

We used Hedegaard et al. (24) and TCGA database as validation cohorts regarding NMIBC and MIBC, respectively. We analyzed the BCa gene expression profile data of NMIBC (Ta, n = 345; T1, n = 112) tissues and MIBC (n = 16) tissues from Hedegaard et al. (24) cohorts. As shown in **Figure 8B**, upregulation of KLHDC7B, CASP14, PRSS1 and downregulation of GAS5 could be also observed in the advanced BCa (T2–4) compared with the early-stage BCa (Ta) (all P < 0.05). However, significantly lower levels of MIR205HG were observed during tumor progression, which was inconsistent with our finding (P < 0.05) (**Figure 8B**). In the TCGA cohort, no statistically significant changes were found in these five RNAs expression levels among advanced BCa (T2: 119, T3: 194, T4: 55) (**Figure 8C**).

### Validating the Prognostic Value of Five Exosomal RNAs in BCa

**Figure 9:**
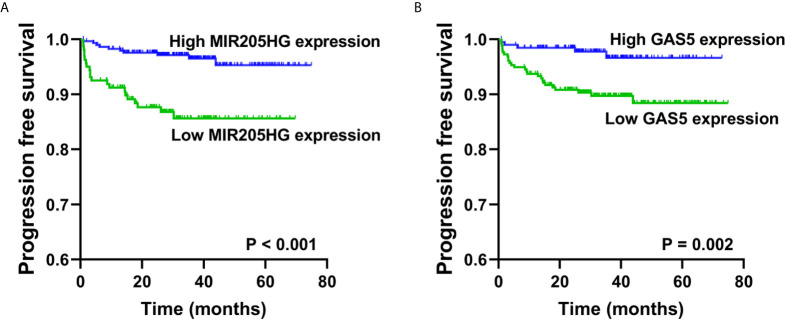
Validation of the prognostic of MIR205HG and GAS5 in NMIBC. Kaplan-Meier curves revealed that low expression of MIR205HG **(A)** and GAS5 **(B)** in tumor tissues were relative to a poor progression free survival in NMIBC patients from Hedegaard et al. cohort.

To further validate the prognostic relevance of these five RNAs in BCa, we used disease progression to MIBC (T2) as the clinical endpoint event for the progression free survival (PFS) of the NMIBC patients, whereas patients’ death for the overall survival (OS) of the MIBC patients. Among five RNAs, ROC curve analyses illustrated that the expression of MIR205HG and GAS5 could predict the progression of NMIBC patients in Hedegaard et al. cohort (P = 0.008, P = 0.002 respectively). The performance of ROC curve analysis and the optimal cut-off value of each RNAs were shown in **Supplementary Table S8**. Based on the optimal cut-off value, we divide the NMIBC patients into the high- or low-expression groups. Kaplan-Meier curves showed that patients with down regulated MIR205HG or GAS5 had a significantly lower PFS (P < 0.001, P = 0.002 respectively, **Figures 9A, B**) compared with their corresponding counterparts. Furthermore, univariate Cox proportional hazards regression showed that age (P = 0.004), tumor stage (P < 0.001), tumor grade (P < 0.001), MIR205HG (P < 0.001), and GAS5 (P = 0.005) were significantly associated with PFS of NMIBC patients, whereas sex (P = 0.624) had no significant influence (**Supplementary Table S9**). Multivariate analysis of the significant influence further indicated that age (HR = 1.044, 95% CI = 1.008–1.081; P = 0.017), tumor stage (HR = 6.783, 95% CI = 3.079–14.943; P < 0.001), MIR205HG (HR = 0.351, 95% CI = 0.163–0.756; P = 0.007), and GAS5 (HR = 0.350, 95% CI = 0.133–0.920; P = 0.033) were independent prognostic factors for the PFS of NMIBC (**Supplementary Table S9**). However, none of the five RNAs can be used to predict the OS of MIBC patients in TCGA cohort (all P > 0.05) (**Supplementary Table S10**).

### Correlation Between Five Exosomal RNAs and Other Clinical Characteristics

To further investigate whether other clinical parameters were related to the expression of these five RNAs in urine exosomes, Spearman’s rank correlation tests were performed in the BCa cases. **Supplementary Table S11** summarizes the relationships between five exosomal RNAs and clinical characteristics of BCa patients in the validation cohort. Only exosomal GAS5 was significantly negatively associated with the tumor stages, tumor grade, and degrees of hematuria (all P < 0.05), whereas, the remaining four exosomal RNAs showed positive correlation with tumor stages, tumor grade, and hematuria degrees (all P < 0.05). However, no significant association were found between five exosomal RNAs and sex or age.

## Discussion

BCa is a heterogeneous disease that characterized by genomic instability and a high mutation rate. Heide et al. (25) performed multi-regional whole-exome sequencing on 10 radical cystectomy specimens from BCa, and the results indicated that a single tumor biopsy specimen may underestimate the mutation burden of heterogeneous tumors. Moreover, they also indicated that mutation genes related to tumor progression, obtained by sequencing of multiple tumor regions, may serve as potential biomarkers to predict disease progression and prognosis at the time of initial diagnosis. In this study, we conducted high-throughput RNA-seq between three matched superficial tumor regions and deep infiltrating tumor regions of MIBC. Eight mRNAs and 32 lncRNAs were significantly upregulated in deep infiltrating tumor regions and were identified as biomarkers related to potential tumor progression. We then further validated the expression levels of selected RNAs in TCGA database, among them 10 RNAs (5 mRNAs and 5 lncRNAs) were differentially expressed in BCa tumor tissues compared with adjacent normal tissues, which could be used as candidate RNAs in subsequent study.

Exosomes derived RNAs, which display both the high specificity of exosomes and the stability of RNAs. Emerging evidence suggests that exosomal RNAs, especially miRNA and lncRNA, can serve as potential biomarkers of BCa. Previous studies have evaluated the presence of urinary exosomes lncRNA as non-invasive biomarkers for BCa cancer detection and recurrences prediction, such as MALAT1, PCAT-1, SPRY4-IT1, UCA1-201, UCA1-203, and LINC00355 (16, 23). However, the performance of the panel (MALAT1, PCAT-1, and SPRY4-IT1) was moderate with an AUC of 0.813 (sensitivity = 62.5%, specificity = 85.0%) in the validation cohort (16). Combination of UCA1-201, UCA1-203, MALAT1, and LINC00355 had excellent AUC of 0.96 and higher sensitivity and specificity of 92.0 and 91.7%, respectively (23). Therefore, RNAs in urine exosomes serve as non-invasive biomarkers, can be used for early diagnosis and prediction of disease progression by avoiding the impact of tumor heterogeneity on single-sample biopsies However, cancer-specific mRNAs that directly affect downstream protein levels and functions have not been well evaluated in exosomes as biomarkers for BCa diagnosis.

Therefore, in this study, 10 candidate RNAs obtained by discovery stage were identified in urine exosomes. After verifying in a large cohort of 80 BCa patients and 80 HCs, we identified three mRNAs (KLHDC7B, CASP14, and PRSS1) and one lncRNA (MIR205HG) that were significantly upregulated in urine exosomes of BCa patients. Whereas, lncRNA, GAS5 was significantly downregulated in urine exosomes of BCa patients compared to HCs, which was inconsistent with the results of TCGA database, considering that the numbers of NMIBC and adjacent normal samples in the TCGA database were limited (NMIBC, n = 4; adjacent normal samples, n = 19) and previous study have demonstrated that GAS5 downregulation was associated with the progression of NMIBC, not MIBC (21), therefore, GAS5 was ultimately retained for subsequent validation. Furthermore, the AUCs of these five exosomal RNAs for BCa diagnosis varied from 0.729 to 0.843. In addition, the combined analysis of these five exosomal RNAs showed a higher diagnostic accuracy with the AUC of 0.924 (sensitivity = 88.5% and specificity = 83.3%), which was higher than any of the single RNAs. Moreover, we demonstrated that the expression levels of these five RNAs were also significantly and consistently differentially expressed between early stage BCa and HCs, the combination of these five RNAs possessed a superior diagnostic capacity (AUC = 0.910; sensitivity = 87.2% and specificity = 83.3%) compared with the use of these molecules alone. These results provided evidence for exosomal RNAs as a new non-invasive diagnostic option for early stage BCa.

KLHDC7B, CASP14, and PRSS1 are putatively oncogenic gene that has been demonstrated in breast cancer (26, 27) and pancreatic cancer (28), and the role of KLHDC7B was correlated with extracellular communication, cell morphology, gene expression, and actin binding (29). MIR205HG is a 4173bp lncRNA, previous study demonstrated that MIR205HG could deplete miR-590-3p leading to proliferation increased-related genes expression in head and neck squamous cell carcinoma (30). However, the evidence for any biological or molecular functions of MIR205HG in disease procession in BCa was limited. GAS5 is a crucial cancer-suppressor lncRNA, and correlated with metastasis in various solid tumors (21, 31–33). Especially in BCa, GAS5 loss was associated with adverse outcome of NMIBC and could be used as a predictor for NMIBC patients’ relapse and progression (21).

In this study, we demonstrated a significant association between urine exosomal RNAs expression and tumor stage which was a vital prognostic feature for BCa patients. We found that upregulation of exosomal KLHDC7B, CASP14, PRSS1, and MIR205HG and downregulation of exosomal GAS5 could be observed in the progression from early-stage BCa (Ta) to MIBC (T2–4). Furthermore, to validate our result in tumor tissues, we further used Hedegaard et al. and TCGA as validation cohorts regarding NMIBC and MIBC, respectively. We found that except for MIR205HG, the results of the other four RNAs mentioned above in exosomes were consistent with those in Hedegaard et al. cohort. Similarly, upregulation of KLHDC7B, CASP14, and PRSS1 and downregulation of GAS5 could be observed in the progression from early-stage (Ta) to advanced BCa (T2–T4). Moreover, MIR205HG and GAS5 expressions were also associated with PFS in patients with NMIBC. In fact, the higher levels of MIR205HG and GAS5 in tissues were associated with decreased BCa progression risk, suggesting that MIR205HG and GAS5 were independent prognostic factors for the PFS of NMIBC. However, there were no statistically significant changes in any of five RNAs levels among advanced BCa (T2–T4) in TCGA database. A possible interpretation of this phenomenon is that the expression of these five RNAs might be a dynamic procedure during the process of carcinoma development, and suggests that these five RNAs may play a biological role in the initiation of tumor muscle invasion, but not in the advanced stage (T2–T4) of tumor. Interestingly, this dual role of KLHDC7B and GAS5 during tumor progression has also been found in other studies. Previous studies on breast cancer provided evidence that KLHDC7B was upregulated in breast tumors, but they also revealed that the expression of KLHDC7B was grade-dependent and only significantly upregulated in grade 3 tumors, indicating that KLHDC7B was associated with more aggressive tumors and worse prognosis (26). Similarly, in MIBC, the decrease of GAS5 expression level was not statistically significant in 63.6% of advanced (T2–T4) patients (21). In conclusion, these five RNAs might be involved in the initiation of muscle invasion and GAS5 could be used as promising novel biomarkers associated with bladder progression in urinary exosomes. The inconsistent expression of MIR205HG in urine exosomes and tissues may be caused by the specificity of specimen origin or the difference in detection methods. However, due to the limited research on the role of MIR205HG in BCa, the specific biological function of MIR205HG in BCa still needs to be further clarified. In addition, the regulatory functions and underlying molecular mechanisms of the other four molecules in BCa development remain unclear. Whether there is physiological significance and what the specific mechanism is also still need further study.

Remarkably, we also found the expression of exosomal GAS5 was significantly negatively associated with the tumor grade and degrees of hematuria (all P < 0.05), whereas, the exosomal KLHDC7B, CASP14, PRSS1, and MIR205HG showed positive correlation with tumor grade and hematuria degrees.

Nevertheless, the current study has several limitations. First, we lack adjacent normal tissues of three matched of samples in the RNA-seq analysis, the use of the TCGA database to verify whether differential RNAs were tumor-related genes was limited. Second, it was unclear whether exosomal RNAs are specific for BCa prognosis, because their expression dynamics were not examined. Third, this study was limited by the lack of validation of candidate RNAs in the corresponding tissues, and larger number of independent cohorts from multi-center will be needed to validate our current findings. Therefore, a multi-center, longitudinal, prospective research in the Chinese population is required to confirm our findings.

## Conclusions

In conclusion, this is the first study to illustrate the combination of exosomal mRNAs and lncRNAs for the diagnosis of BCa and we identified five molecules as promising biomarkers for an early stage BCa diagnosis. Further multi-center studies are needed to confirm the current findings.

## Data Availability Statement

The datasets presented in this study can be found in online repositories. The names of the repository/repositories and accession number can be found below: the NCBI Gene Expression Omnibus (GSE172359).

## Ethics Statement

The studies involving human participants were reviewed and approved by the Ethics Committee of Peking University First Hospital. The patients/participants provided their written informed consent to participate in this study.

## Author Contributions

HL, WY, HH, and HmH contributed to conception and design of the study. HH and HmH collected the tissues and urine samples as well as their clinical information. HmH, JD, and JB carried out the experiments. HmH and LP organized the database. ND, CH, and JH performed the data analysis. HmH wrote the manuscript. HL, WY, and HH revised the manuscript. All authors contributed to the article and approved the submitted version.

## Conflict of Interest

The authors declare that the research was conducted in the absence of any commercial or financial relationships that could be construed as a potential conflict of interest.
