# Supplementary material for: Combination of Urine Exosomal mRNAs and lncRNAs as Novel Diagnostic Biomarkers for Bladder Cancer
Source: Front Oncol. 2021 Apr 27;11:667212. doi: 10.3389/fonc.2021.667212 (PMC8111292; doi:10.3389/fonc.2021.667212)
Supplement: Supplementary file 1 [file DataSheet_1.docx]

Supplementary Material

## Supplementary Figures


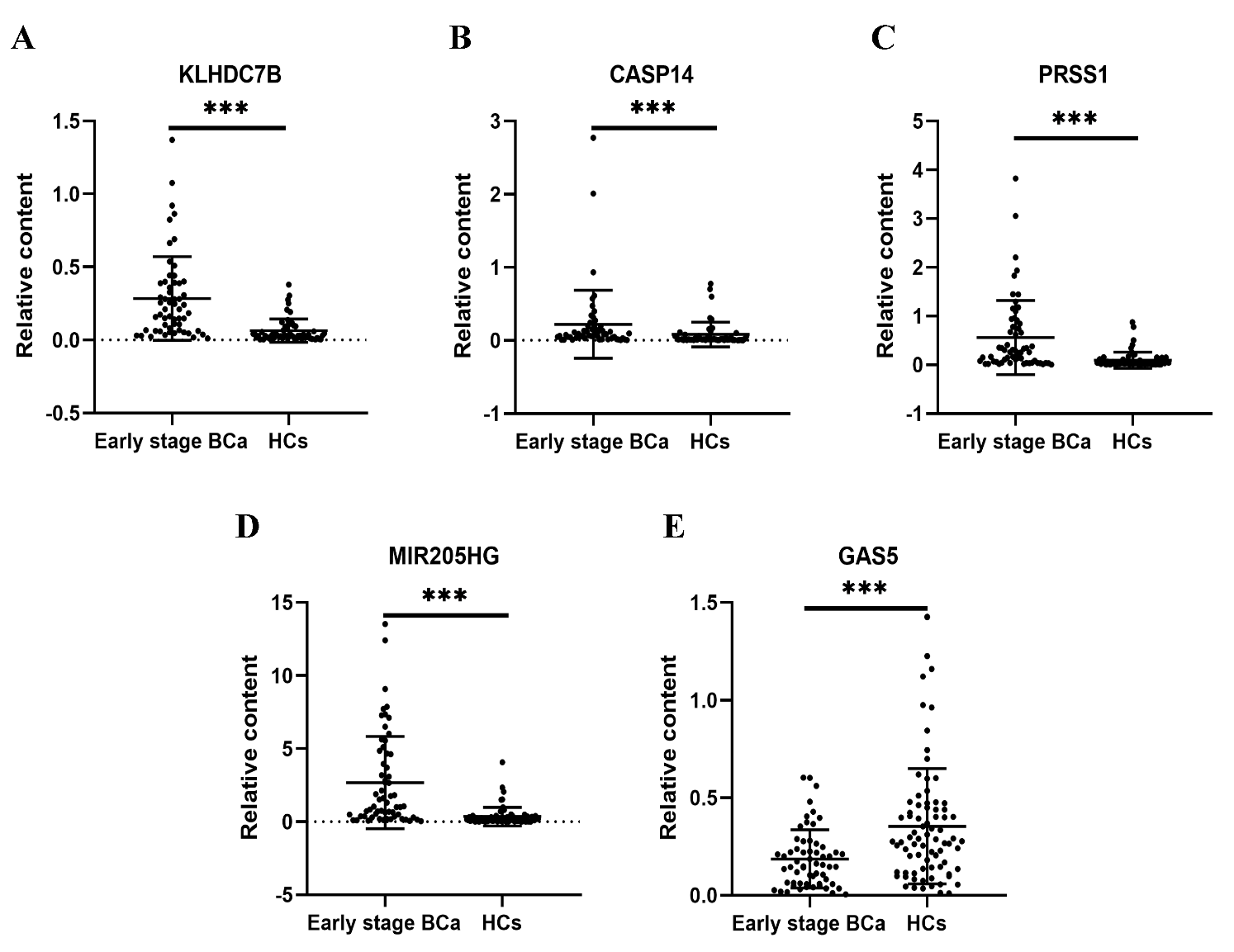


**Figure S1. The relative expression of urine exo-RNAs in early stage bladder cancer (BCa) patients (n = 65) and healthy controls (HCs) (n = 80) using real-time quantitative PCR (RT-qPCR).** (A) Relative expression of urine exo-KLHDC7B. (B) Relative expression of urine exo-CASP14. (C) Relative expression of urine exo-PRSS1. (D) Relative expression of urine exo-MIR205HG. (E) Relative expression of urine exo-GAS5. data was shown as mean ± 95% CI. *** represents P < 0.001.
