# Supplementary material for: Combination of Urine Exosomal mRNAs and lncRNAs as Novel Diagnostic Biomarkers for Bladder Cancer
Source: Front Oncol. 2021 Apr 27;11:667212. doi: 10.3389/fonc.2021.667212 (PMC8111292; doi:10.3389/fonc.2021.667212)
Supplement: Supplementary file 2 [file DataSheet_2.docx]

Supplementary Methods

**RNA sequencing data analysis**

1. **Data preprocessing and genomic alignment**

Raw reads generated during high-throughput sequencing were fastq format sequences. Raw reads are further quality filtered to obtain high-quality reads that could be used for later analysis. Trimmomatic ([Bolger et al., 2014](#_ENREF_2)) software was first used for adpter removing, And then low-quality bases and N-bases or low-quality reads were filtered out. Finally, we conducted quality detection through FastQC software to ensure the reliability of the obtained reads. And then we got high-quality clean reads.

Clean reads with certain range of length were mapped to reference sequence using hisat2 ([Kim et al., 2015](#_ENREF_3)) [2] from Genome Database (GRCh38.p12) <ftp://ftp.ncbi.nlm.nih.gov/genomes/all/GCF/000/001/405/GCF_000001405.38_GRCh38.p12/GCF_000001405.38_GRCh38.p12_genomic.fna.gz>

and mRNA Database <ftp://ftp.ncbi.nlm.nih.gov/genomes/all/GCF/000/001/405/GCF_000001405.38_GRCh38.p12/GCF_000001405.38_GRCh38.p12_rna.fna.gz>

1. **Transcript splicing, lncRNA prediction, and Gene quantification**

The result of alignment with the reference genome was stored in a binary file, called a bam file. Using the Stringtie ([Pertea et al., 2015](#_ENREF_7)) software to assemble the reads, the new transcript was spliced. Then the candidate lncRNA transcripts were selected by comparing the gene annotation information of the reference sequence produced by Cuffcompare ([Trapnell et al., 2012](#_ENREF_11)) software. Finally, transcripts with coding potential were screened out by CPC ([Kong et al., 2007](#_ENREF_4)) , CNCI ([Sun et al., 2013](#_ENREF_10)) , Pfam ([Sonnhammer et al., 1998](#_ENREF_9)) and PLEK([Li et al., 2014](#_ENREF_6)) to obtain lncRNA predicted sequences.

Aligning the sequencing reads of each sample with the sequence of mRNA transcript sequences, known lncRNA sequences and lncRNA prediction sequences by bowtie2([Langmead and Salzberg, 2012](#_ENREF_5)), and using eXpress([Roberts and Pachter, 2013](#_ENREF_8)) to make gene quantitative analysis, the fragments per kilobase of model per million base pairs sequenced (FPKM) was used to calculate the expression levels of mRNA or lncRNA. the FPKM value was calculated as follows:

$$FPKM=\frac{F\mathrm{ragments} mapped to the transcript}{\mathrm{Total}\mathrm{fragments}\times Transcript length}\times{10}^{9}$$

1. **Differential screening analysis and Functional Analysis**

Using the estimateSizeFactors function of the DESeq R package (version 1.8.3) to normalize the counts, and using nbinomTest function to calculate P value and flod change values for the difference comparison. Finally, the differential expression (DE) RNAs with P < 0.05 and fold change > 2 were identified.

The function and biological pathways of differentially expressed RNAs were analyzed with Gene Ontology (GO) (http://www.geneontology.org) and Kyoto Encyclopedia of Genes and Genomes (KEGG) (http://www.genome.ad.j/kegg/) databases by using Hypergeometric Distribution Test. GO analysis can be divided into molecular function, biological process and cellular component. Each GO term or KEGG pathway was taken as a functional module, and the significance of each differential mRNA/lncRNA being enriched in GO or KEGG was tested through Hypergeometric Distribution Test. The calculation formula is as follows:

$$p=1-\sum_{i=0}^{m-1} \frac{\binom{M}{i}\binom{N-M}{n-i}}{\binom{N}{n}}$$

Where N is the number of mRNAs/lncRNAs with GO (KEGG) annotation in all mRNAs/lncRNAs; n is the number of differentially expressed (DE) mRNAs/lncRNAs with GO (KEGG) annotation; M is the number of mRNAs/lncRNAs annotated with a specific GO term (KEGG pathway) among all mRNAs/lncRNAs; m is the number of DE mRNAs/lncRNAs annotated as a specific GO term (KEGG pathway). P value < 0.05 were considered to be significantly enriched.

Supplementary Tables

| **Table S1. Primers sequences used for RT-qPCR.** | | |
| --- | --- | --- |
| ***Gene Symbol*** | **Forward primer** | **Reverse primer** |
| *KLHDC7B* | 5' GCCCTGCAACCGAAGTCTAA 3' | 5' ACAGCCGTCTTGAGTGGAAG 3' |
| *CASP14* | 5' ACCATGAAAAGAGACCCCACT 3' | 5' GAGTACCACGAAGGCACAACT 3' |
| *ESM1* | 5' ACCTTCGGGATGGATTGCAG 3' | 5' GATGCCATGTCATGCTCCGT 3' |
| *PRSS1* | 5' CTTCTCCTGGGACTAGGCTCTGT 3' | 5' TGTCCAGGTTGCTGACGGTA 3' |
| *CST1* | 5' CCTGTGCCTTCCATGAACAGCC 3' | 5' GGGTGGTGGCTGGTGCCAATG 3' |
| *GAS5* | 5' TGTGTCCCCAAGGAAGGATG 3' | 5' TCCACACAGTGTAGTCAAGCC 3' |
| *PVT1* | 5' CTCTGCTGTGGCTGCCC 3' | 5' GGGTGGGAAAGAGAGGTTCG 3' |
| *MIR205HG* | 5' GGAGTGCAGTGGCTCAATCT 3' | 5' TGGATTGCTTAAGCTCAGGA 3' |
| *SNHG12* | 5' TTCAAGCGATTCTCGTGCC 3' | 5' AAGATTGTCAAACCCTCCCTGT 3' |
| *LINC02474* | 5‘-TGTTTCCCAAAGTGGCACCT-3' | 5‘-ACGCACACAATCTACCCCTG-3' |

| **Table S2 . Up-regulated mRNAs in RNA-seq and TCGA cohort.** | | | | | | | | | | |  |
| --- | --- | --- | --- | --- | --- | --- | --- | --- | --- | --- | --- |
| **Gene_id** |  | **RNA-seq** | | | | | |  | **TCGA cohort** | | |
|  |  | **High expression in deep infiltrating tumor regions (vs superficial tumor regions )** | | | | | |  | **High expression in MIBC tissues (vs adjacent normal tissues )** | | |
|  |  | **FC** | **Log_2_FC** | **Expression_superficial tumor regions** | **Expression_deep infiltrating tumor regions** | **Regulation** | **P Value** |  | **Regulation** | **P Value** | **FDR adjusted P value** |
| LEFTY1 |  | 3.777 | 1.917 | 0.844 | 3.895 | Up | 0.018 |  | Not | 0.745 | 0.813 |
| CASP14 |  | 3.196 | 1.676 | 11.398 | 14.4 | Up | 0 |  | Up | <0.001 | <0.001 |
| PRSS1 |  | 2.847 | 1.51 | 1.951 | 7.635 | Up | 0.026 |  | Up | 0.004 | 0.011 |
| KLHDC7B |  | 2.788 | 1.479 | 30.542 | 34.982 | Up | 0 |  | Up | <0.001 | < 0.001 |
| B3GALT5 |  | 2.67 | 1.417 | 1.841 | 5.414 | Up | 0.001 |  | Not | 0.522 | 0.626 |
| CST1 |  | 2.396 | 1.26 | 1.947 | 6.42 | Up | 0.035 |  | Up | <0.001 | < 0.001 |
| ESM1 |  | 2.09 | 1.064 | 4.029 | 7.71 | Up | 0 |  | Up | <0.001 | < 0.001 |
| TFF1 |  | 2.014 | 1.01 | 8.845 | 22.845 | Up | 0.045 |  | Not | 0.816 | 0.851 |
| RNA-seq, RNA sequencing; TCGA, The Cancer Genome Atlas; FC, Foldchange; FDR, the false discovery rate | | | | | | | | | | | |

| **Table S3 . Up-regulated lncRNAs in RNA-seq and TCGA cohort.** | | | | | | | | | | | |  |
| --- | --- | --- | --- | --- | --- | --- | --- | --- | --- | --- | --- | --- |
| **Gene_id** |  | **RNA-seq** | | | | | |  | **TCGA cohort** | | | |
|  |  | **High expression in deep infiltrating tumor regions (vs superficial tumor regions )** | | | | | |  | **High expression in MIBC tissues (vs adjacent normal tissues )** | | | |
|  |  | **FC** | **Log_2_FC** | **Expression_superficial tumor regions** | **Expression_deep infiltrating tumor regions** | **Regulation** | **P Value** |  | **Regulation** | **P Value** | **FDR adjusted P value** | |
| XLOC_000512 |  | 321.040 | 8.327 | 0.000 | 8.206 | Up | 0.033 |  | - | - | - | |
| XLOC_016150 |  | 218.370 | 7.771 | 2.644 | 7.433 | Up | 0.008 |  | - | - | - | |
| GAS5 |  | 184.771 | 7.530 | 0.000 | 3.321 | Up | 0.000 |  | UP | 0.028 | 0.048 | |
| EPB41L4A-AS1 |  | 125.647 | 6.973 | 0.028 | 3.731 | Up | 0.000 |  | Down | 0.016 | 0.032 | |
| XLOC_015920 |  | 83.408 | 6.382 | 0.000 | 3.914 | Up | 0.003 |  | - | - | - | |
| AP001120.2 |  | 82.475 | 6.366 | 0.170 | 14.907 | Up | 0.000 |  | Not | 0.107 | 0.171 | |
| XLOC_005533 |  | 63.895 | 5.998 | 0.453 | 3.540 | Up | 0.012 |  | - | - | - | |
| DLGAP1-AS1 |  | 30.740 | 4.942 | 0.313 | 3.753 | Up | 0.000 |  | Not | 0.383 | 0.484 | |
| SNHG12 |  | 22.069 | 4.464 | 3.706 | 4.579 | Up | 0.002 |  | Up | <0.001 | <0.001 | |
| XLOC_011443 |  | 19.985 | 4.321 | 2.964 | 13.719 | Up | 0.000 |  | - | - | - | |
| PVT1 |  | 16.742 | 4.065 | 0.476 | 4.442 | Up | 0.003 |  | Up | <0.001 | < 0.001 | |
| LOC105378938 |  | 15.759 | 3.978 | 0.904 | 3.032 | Up | 0.001 |  | - | - | - | |
| NUTM2A-AS1 |  | 9.024 | 3.174 | 1.188 | 5.740 | Up | 0.007 |  | Not | 0.163 | 0.245 | |
| XLOC_000091 |  | 7.193 | 2.847 | 41.325 | 380.829 | Up | 0.000 |  | - | - | - | |
| LRRC75A-AS1 |  | 6.487 | 2.698 | 2.002 | 5.687 | Up | 0.004 |  | - | - | - | |
| SMILR |  | 5.807 | 2.538 | 1.071 | 3.655 | Up | 0.027 |  | Not | 0.704 | 0.805 | |
| LINC02474 |  | 5.737 | 2.520 | 1.650 | 3.203 | Up | 0.044 |  | Up | <0.001 | < 0.001 | |
| AL121790.2 |  | 5.707 | 2.513 | 3.514 | 8.625 | Up | 0.009 |  | Not | 0.201 | 0.268 | |
| XLOC_013034 |  | 5.693 | 2.509 | 1.066 | 4.068 | Up | 0.033 |  | - | - | - | |
| XLOC_015017 |  | 4.814 | 2.267 | 23.488 | 26.751 | Up | 0.043 |  | - | - | - | |
| XLOC_006876 |  | 4.414 | 2.142 | 16.509 | 33.511 | Up | 0.008 |  | - | - | - | |
| AC019117.1 |  | 4.232 | 2.082 | 1.106 | 4.989 | Up | 0.025 |  | Not | 0.181 | 0.256 | |
| MIR205HG |  | 3.140 | 1.651 | 1.631 | 4.462 | Up | 0.017 |  | Up | 0.006 | 0.013 | |
| TCONS_00022025 |  | 3.060 | 1.614 | 17.551 | 30.333 | Up | 0.020 |  | - | - | - | |
| UCA1 |  | 2.980 | 1.575 | 28.730 | 72.815 | Up | 0.005 |  | Up | 0.018 | 0.033 | |
| AC048344.4 |  | 2.774 | 1.472 | 3.484 | 9.403 | Up | 0.015 |  | Not | 0.975 | 0.975 | |
| TUG1 |  | 2.683 | 1.424 | 9.033 | 17.866 | Up | 0.013 |  | Up | 0.002 | 0.006 | |
| XLOC_000516 |  | 2.635 | 1.398 | 15.694 | 35.299 | Up | 0.006 |  | - | - | - | |
| DHRS4-AS1 |  | 2.517 | 1.332 | 1.898 | 3.577 | Up | 0.011 |  | - | - | - | |
| XLOC_001865 |  | 2.480 | 1.311 | 12.647 | 26.869 | Up | 0.017 |  | - | - | - | |
| AC093157.1 |  | 2.480 | 1.310 | 1.629 | 3.139 | Up | 0.034 |  | - | - | - | |
| LOXL1-AS1 |  | 2.346 | 1.230 | 2.243 | 3.738 | Up | 0.039 |  | Down | 0.005 | 0.012 | |
| RNA-seq, RNA sequencing; TCGA, The Cancer Genome Atlas; FC, Foldchange; FDR, the false discovery rate | | | | | | | | | | | |  |

| **Table S4. The relative concentrations of candidate mRNAs confirmed by RT-qPCR in urine exosomes from the BCa patients and healthy controls (HCs) in the training cohort (n=20).** | | | | |
| --- | --- | --- | --- | --- |
| **mRNA** | **BCa** | **HCs** | ***P*-value** | **Results** |
| **CASP14** | 0.166 (0.06) | 0.015 (0.004) | 0.001 | significant |
| **PRSS1** | 0.416 (0.128) | 0.020 (0.004) | <0.001 | significant |
| **KLHDC7B** | 0.407 (0.097) | 0.033 (0.013) | <0.001 | significant |
| **CST1** | 0.061 (0.032) | 0.013 (0.001) | 0.497 | non-significant |
| **ESM1** |  |  |  | Ct value >35 |
| mRNA data are expressed as mean (SE) | | |  |  |

| **Table S5. The relative concentrations of candidate lncRNAs confirmed by RT-qPCR in urine exosomes from the BCa patients and healthy controls (HCs) in the training cohort (n=20).** | | | | |
| --- | --- | --- | --- | --- |
| **lncRNA** | **BCa** | **HCs** | ***P*-value** | **Results** |
| **SNHG12** |  |  |  | Ct value >35 |
| **PVT1** |  |  |  | Ct value >35 |
| **LINC02474** |  |  |  | Ct value >35 |
| **MIR205HG** | 3.278 (1.041) | 0.112 (0.035) | <0.001 | significant |
| **GAS5** | 0.114 (0.03) | 0.380 (0.092) | 0.002 | significant |
| lncRNA data are expressed as mean (SE) | | |  |  |

| **Table S6: Multiple regression analysis of the associations between five exosomal RNAs and the incidence of BCa in validation cohort.** | | | | |
| --- | --- | --- | --- | --- |
| **Model 1: KLHDC7B, R^2^=0.445** | | | | |
|  | **B** | **SE** | **OR (95% CI)** | **P value** |
| **KLHDC7B^a^** | 0.004 | 0.001 | 1.004 (1.001 - 1.007) | 0.006 |
| **Age** | 0.115 | 0.023 | 1.122 (1.072 - 1.174) | <0.001 |
|  |  |  |  |  |
| **Model 2: CASP14, R^2^=0.438** | | | | |
|  | **B** | **SE** | **OR (95% CI)** | **P value** |
| **CASP14^a^** | 0.002 | 0.001 | 1.002 (1.000 - 1.005) | 0.046 |
| **Age** | 0.143 | 0.027 | 1.153 (1.094-1.216) | <0.001 |
|  |  |  |  |  |
| **Model 3: PRSS1, R^2^=0.482** | | | | |
|  | **B** | **SE** | **OR (95% CI)** | **P value** |
| **PRSS1^a^** | 0.003 | 0.001 | 1.003 (1.001 - 1.005) | 0.003 |
| **Age** | 0.125 | 0.025 | 1.133 (1.079 - 1.190) | <0.001 |
|  |  |  |  |  |
| **Model 4: MIR205HG, R^2^=0.479** | | | | |
|  | **B** | **SE** | **OR (95% CI)** | **P value** |
| **MIR205HG** | 0.441 | 0.123 | 1.554 (1.221 - 1.976) | <0.001 |
| **Age** | 0.133 | 0.025 | 1.142 (1.088 - 1.200) | <0.001 |
|  |  |  |  |  |
| **Model 5: GAS5, R^2^=0.396** | | | | |
|  | **B** | **SE** | **OR (95% CI)** | **P value** |
| **GAS5** | -3.318 | 1.209 | 0.036 (0.003 - 0.387) | 0.006 |
| **Age** | 0.119 | 0.022 | 1.126 (1.079-1.175) | <0.001 |
| SE, standard error; OR, odds ratio; CI, confidence interval | | | | |
| ^a^ The original value of KLHDC7B, CASP14 and PRSS1 has been multiplied by 1000, because the OR value obtained from the original value is too large | | | | |

| **Table S7. Comparison of the ROC curves of single RNA and combined RNAs panel by DeLong test in validation cohort (P value).** | | | | | | | |
| --- | --- | --- | --- | --- | --- | --- | --- |
| **RNAs combination** | **KLHDC7B** | **CASP14** | **PRSS1** | **MIR205HG** | **GAS5** | **mRNAs panel^a^** | **lncRNAs panel^b^** |
| **mRNAs panel^a^** | 0.380 | 0.011* | 0.207 | 0.356 | 0.142 | 1.000 | 0.681 |
| **lncRNAs panel^b^** | 0.359 | 0.0149* | 0.273 | 0.237 | 0.004** | 0.681 | 1.000 |
| **Five RNAs panel^c^** | 0.0246* | 0.001** | 0.0113* | 0.0192* | <0.001*** | 0.027* | 0.048* |
| ROC, receiver operating characteristic | | | | | | | |
| a: mRNAs panel: KLHDC7B, CASP14, PRSS1 | | | | | | | |
| b: lncRNAs panel: MIR205HG, GAS5 | | | | | | | |
| c: Five RNAs panel: KLHDC7B, CASP14, PRSS1,MIR205HG, GAS5  *** P < 0.001, ** P < 0.01, * P < 0.05 | | | | | | | |
|  | | | | | | | |

| **Table S8. Prognostic performance of five RNAs for predicting the progression of NMIBC patients (Ta, n = 345; T1, n = 112) in Hedegaard et al cohort.** | | | | | |
| --- | --- | --- | --- | --- | --- |
| **RNAs** | **AUC (95% CI)** | **P value** | **Sensitivity%** | **Specificity%** | **Optimal cut-off values** |
| **KLHDC7B** | 0.547 (0.429 - 0.665) | 0.060 |  |  |  |
| **CASP14** | 0.552 (0.442 - 0.662) | 0.056 |  |  |  |
| **PRSS1** | 0.558 (0.446 - 0.670) | 0.057 |  |  |  |
| **MIR205HG** | 0.642 (0.521 - 0.763) | 0.008 | 67.7 | 66.7 | 91.876 |
| **GAS5** | 0.667 (0.567 - 0.768) | 0.002 | 83.9 | 45.5 | 67.597 |

| **Table S9. Univariate and multivariate Cox proportional hazards regression model analysis of progression-free survival in patients with NMIBC (Ta, n = 345; T1, n = 112) from Hedegaard et al. cohort.** | | | | | | |
| --- | --- | --- | --- | --- | --- | --- |
|  |  | **Progression-free survival** | | | | |
|  |  | **Univariate analysis** | |  | **Multivariate analysis** | |
| **Covariant** | **Categories** | **HR (95% CI)** | **P value** |  | **HR (95% CI)** | **P value** |
| **Age (continuous variable)** |  | 1.056 (1.017 - 1.096) | 0.004 |  | 1.044 (1.008 - 1.081) | 0.017 |
| **Sex** | Female vs Male | 0.818 (0.366 - 1.828) | 0.624 |  |  |  |
| **Tumor stage** | T1 vs Ta | 9.123 (4.195 - 19.842) | < 0.001 |  | 6.783 (3.079 - 14.943) | < 0.001 |
| **Tumor grade** | High vs Low | 4.516 (2.078 - 9.815) | < 0.001 |  |  |  |
| **MIR205HG** | High vs Low | 0.235 (0.110 - 0.499) | < 0.001 |  | 0.351 (0.163 - 0.756) | 0.007 |
| **GAS5** | High vs Low | 0.253 (0.097 - 0.660) | 0.005 |  | 0.350 (0.133 - 0.920) | 0.033 |

| **Table S10. Prognostic performance of five RNAs for predicting the overall survival of MIBC patients (T2: 119, T3: 194, T4: 55) in TCGA cohort** | | |
| --- | --- | --- |
| **RNAs** | **AUC (95% CI)** | **P value** |
| **KLHDC7B** | 0.544 (0.485 - 0.604) | 0.144 |
| **CASP14** | 0.524 (0.464 - 0.583) | 0.435 |
| **PRSS1** | 0.513 (0.454 - 0.573) | 0.695 |
| **MIR205HG** | 0.553 (0.493 - 0.613) | 0.081 |
| **GAS5** | 0.537 (0.477 - 0.596) | 0.228 |

| **Table S11. The results of Spearman’s rank correlations between five urine exosomal RNAs and clinical characteristics in BCa patients (n=80).** | | | | | |
| --- | --- | --- | --- | --- | --- |
| **Variables** | **KLHDC7B** | **CASP14** | **PRSS1** | **MIR205HG** | **GAS5** |
| **Sex** | *0.011* | *0.108* | *0.151* | *0.103* | *-0.032* |
|  | *P=0.928* | *P=0.369* | *P=0.203* | *P=0.382* | *P=0.783* |
| **Age** | *-0.021* | *-0.015* | *0.042* | *0.035* | *-0.017* |
|  | *P=0.865* | *P=0.905* | *P=0.728* | *P=0.770* | *P=0.884* |
| **Tumor stage** | *0.393^**^* | *0.469^**^* | *0.309^**^* | *0.407^**^* | *-0.347*** |
|  | *0.001* | *P<0.001* | *P=0.008* | *P<0.001* | *P=0.002* |
| **Tumor grade** | *0.406^***^* | *0.451^***^* | *0.334^**^* | *0.438^***^* | *-0.285** |
|  | *P<0.001* | *P<0.001* | *P=0.004* | *P<0.001* | *P=0.013* |
| **Hematuria degree** | *0.361*** | *0.357*** | *0.453**** | *0.414**** | *-0.338*** |
|  | *P=0.002* | *P=0.003* | *P<0.001* | *P<0.001* | *P=0.004* |

*** P < 0.001, ** P < 0.01, * P < 0.05

**Reference**

*FastQC: A quality control tool for high throughput sequence data.* [Online]. Available: <https://www.bioinformatics.babraham.ac.uk/projects/fastqc/> [Accessed].

Bolger, A.M., Lohse, M., and Usadel, B. (2014). Trimmomatic: a flexible trimmer for Illumina sequence data. *Bioinformatics (Oxford, England).* 30(15)**,** 2114-2120. doi: 10.1093/bioinformatics/btu170

Kim, D., Langmead, B., and Salzberg, S.L. (2015). HISAT: a fast spliced aligner with low memory requirements. *Nature methods.* 12(4)**,** 357-360. doi: 10.1038/nmeth.3317

Kong, L., Zhang, Y., Ye, Z.-Q., Liu, X.-Q., Zhao, S.-Q., Wei, L., et al. (2007). CPC: assess the protein-coding potential of transcripts using sequence features and support vector machine. *Nucleic acids research.* 35(Web Server issue)**,** W345-W349. doi: 10.1093/nar/gkm391.

Langmead, B., and Salzberg, S.L. (2012). Fast gapped-read alignment with Bowtie 2. *Nature methods.* 9(4)**,** 357-359. doi: 10.1038/nmeth.1923

Li, A., Zhang, J., and Zhou, Z. (2014). PLEK: a tool for predicting long non-coding RNAs and messenger RNAs based on an improved k-mer scheme. *BMC bioinformatics.* 15**,** 311. doi: 10.1186/1471-2105-15-311

Pertea, M., Pertea, G.M., Antonescu, C.M., Chang, T.-C., Mendell, J.T., and Salzberg, S.L. (2015). StringTie enables improved reconstruction of a transcriptome from RNA-seq reads. *Nature biotechnology.* 33(3)**,** 290-295. doi: 10.1038/nbt.3122

Roberts, A., and Pachter, L. (2013). Streaming fragment assignment for real-time analysis of sequencing experiments. *Nature methods.* 10(1)**,** 71-73. doi: 10.1038/nmeth.2251

Sonnhammer, E.L., Eddy, S.R., Birney, E., Bateman, A., and Durbin, R. (1998). Pfam: multiple sequence alignments and HMM-profiles of protein domains. *Nucleic acids research.* 26(1)**,** 320-322. doi: 10.1093/nar/26.1.320.

Sun, L., Luo, H., Bu, D., Zhao, G., Yu, K., Zhang, C., et al. (2013). Utilizing sequence intrinsic composition to classify protein-coding and long non-coding transcripts. *Nucleic acids research.* 41(17)**,** e166. doi: 10.1093/nar/gkt646

Trapnell, C., Roberts, A., Goff, L., Pertea, G., Kim, D., Kelley, D.R., et al. (2012). Differential gene and transcript expression analysis of RNA-seq experiments with TopHat and Cufflinks. *Nature protocols.* 7(3)**,** 562-578. doi: 10.1038/nprot.2012.016
